# Construction and analysis of competitive endogenous RNA networks and prognostic models associated with ovarian cancer based on the exoRBase database

**DOI:** 10.1371/journal.pone.0291149

**Published:** 2024-04-11

**Authors:** Zanhao Chen, Chongyu Wang, Jianing Ding, Tingting Yu, Na Li, Cong Ye

**Affiliations:** 1 Department of Medicine, Xinglin College, Nantong University, Nantong City, Jiangsu Province, China; 2 Department of Gynecology, Taicang Affiliated Hospital of Soochow University (The First People’s Hospital of Taicang), Suzhou City, Jiangsu Province, China; Xiangya Hospital Central South University, CHINA

## Abstract

**Objective:**

To construct a competitive endogenous RNA (ceRNA) regulatory network in blood exosomes of patients with ovarian cancer (OC) using bioinformatics and explore its pathogenesis.

**Methods:**

The exoRbase2.0 database was used to download blood exosome gene sequencing data from patients OC and normal controls and the expression profiles of exosomal mRNA, long non-coding RNA (lncRNA), and circular RNA (circRNA) were detected independently using R language for differential expression analysis. TargetScan and miRanda databases were combined for the prediction and differential expression of mRNA-binding microRNAs (miRNA). The miRcode and starBase databases were used to predict miRNAs that bind to differentially expressed lncRNAs and circRNAs repectively. The relevant mRNA, circRNA, lncRNA and their corresponding miRNA prediction data were imported into Cytoscape software for visualization of the ceRNA network. The R language and KEGG Orthology-based Annotation System (KOBAS) were used to execute and illustrate the Gene Ontology (GO) and Kyoto Encyclopedia of Genes and Genomes (KEGG) enrichment analyses. Hub genes were identified using The CytoHubba plugin.

**Results:**

Thirty-one differentially expressed mRNAs, 17 differentially expressed lncRNAs, and 24 differentially expressed circRNAs were screened. Cytoscape software was used to construct the ceRNA network with nine mRNA nodes, two lncRNA nodes, eight circRNA nodes, and 51 miRNA nodes. Both GO and KEGG were focused on the Spliceosome pathway, indicating that spliceosomes are closely linked with the development of OC, while *heterogenous nuclear ribonucleoprotein K* and *RNA binding motif protein X-linked* genes were the top 10 score Hub genes screened by Cytoscape software, including two lncRNAs, four mRNAs, and four circRNAs. In patients with OC, the expression of *eukaryotic translation initiation factor 4 gamma 2* (EIF4G2), *SERPINE 1 mRNA binding protein 1* (SERBP1), *ribosomal protein L15* (RPL15) and *human leukocyte antigen complex P5* (HCP5) was significantly higher whereas that of *testis expressed transcript*, *Y-linked 15* and *DEAD-box helicase 3 Y-linked* genes was lower compared to normal controls Immunocorrelation scores revealed that SERBP1 was significantly and negatively correlated with endothelial cells and CD4+ T cells and positively correlated with natural killer (NK) cells and macrophages, respectively; RPL15 was significantly positively correlated with macrophages and endothelial cells and negatively correlated with CD8+ T cells and uncharacterized cells, respectively. *EIF4G2* was significantly and negatively correlated with endothelial cells and CD4+ T cells, and positively correlated with uncharacterized cells, respectively. Based on the survival data and the significant correlation characteristics derived from the multifactorial Cox analysis (*P < 0*.*05*), the survival prediction curves demonstrated that the prognostic factors associated with 3-year survival in patients with OC were The prognostic factors associated with survival were Macrophage, RPL15.

**Conclusion:**

This study successfully constructs a ceRNA regulatory network in blood exosomes of OV patients, which provides the specific targets for diagnosis and treatment of OC.

## 1 Introduction

Ovarian cancer (OC) is one of the most prevalent and dangerous malignancies affecting the female reproductive system. It is commonly referred to as the "king of female cancer" attributed to its high incidence and significant impact on female health and well-being. Global statistics demonstrate that every year, approximately 240,000 females are diagnosed with ovarian cancer of whom, tragically, about 140,000 lose their lives [1,2]. The available clinical treatments for ovarian cancer are limited, primarily comprising surgery, chemotherapy, or targeted drug therapy. However, the curative potential of surgery alone is limited to a considerably small number of patients, and the vast majority of patients typically require comprehensive treatment, such as a combination of surgery and chemotherapy. However, patients with OC have insidious clinical symptoms in the early stage, and over 75% of them are already in the advanced stage (FIGO stage III or IV) when they are diagnosed, and the 5-year survival rate of these patients is <25% [3,4]. When OC is diagnosed at an early stage, such as FIGO stage I or II, and prompt treatment is provided, the 5-year survival rate of patients can increase to 90% [5]. Despite the improvement of conventional clinical treatments, recurrence of OC and chemotherapy resistance remain issues. Thus, investigating the underlying mechanisms of OC initiation, progression, invasion, and metastasis, and identifying early diagnostic markers and therapeutic targets with enhanced specificity and sensitivity are crucial objectives in impeoving the patients with OC.

Exosomes are circular vesicles with a lipid bilayer and a diameter of 40–100 nm. They are secreted by various active cells and abundantly present in different body fluids, including saliva, serum, plasma, and urine [6,7]. OC cell-derived exosomes, which transfer long non-coding RNA (lncRNA), microRNA (miRNA)and some biologically active substances, play an important role in the tumor microenvironment, by altering the biochemical composition, signaling pathways, and gene regulation of recipient cells, thus, participating in the development of OC proliferation, invasion, metastasis, immune evasion, and chemotherapy resistance [8,9]. Studies have confirmed that exosome miR-21 is involved in malignant transformation in serious OC by targeting the tumor suppressor PDCD4 [10]. In serous OC effusions, exosomes maintain overexpression of miR-21 and deletion of PDCD4, which may potentially contribute to tumor spread [11,12]. In 2011, Salmena et al. introduced the competitive endogenous RNA (ceRNA) hypothesis. According to this hypothesis, within the ceRNA network, regulatory interactions exist among mRNA, lncRNA, circular RNA (circRNA), and all other RNA transcripts. These interactions involve competition for miRNA response elements, allowing these transcripts to regulate each other’s expression. Consequently, this regulatory mechanism further influences various biological processes such as tumor cell proliferation, growth, differentiation, and apoptosis [13]. Existing studies suggest that ceRNAs are involved in OC development, however, their regulatory mechanism in blood exosomes of patients with OC remains elusive [14].

In this study, we analyzed blood exosome sequencing data obtained from patients with OC and normal controls using the exoRBase database. Using this analysis, we identified differential expression profiles of mRNA, lncRNA, and circRNA. By constructing ceRNA networks, we aimed to provide a theoretical foundation for the exploration of novel targets in the diagnosis and treatment of OC. This research may contribute to the development of more effective and targeted approaches to combat OC, ultimately improving patient outcomes.

## 2 Materials and methods

### 2.1 Data installation and screening of differentially expressed mRNA, lncRNA, and circRNA

Exosome gene sequencing data of blood samples from patients with OC and normal controls were obtained from the exoRBase 2.0 database (http://www.exorbase.org/). The data cut-off date was August 29, 2022. A total of 148 sample sets were included, comprising 30 sets of exosome gene sequencing data from patients with OC and 118 sets from normal samples, respectively. Corresponding gene annotation files were also downloaded from exoRbase. In this study, the experimental group comprised blood exosomes from patients with OC, while the control group comprised exosomes from normal samples. The expression profiles of mRNA, lncRNA, and circRNA in exosomes were subjected to differential expression analysis using the screening condition of |log2 FC| > 0 and the corrected screening condition of P < 0.05.

### 2.2 Interaction, RNA prediction, and construction of ceRNA network

The TargetScan (http://www.targetscan.org/vert_71/) and miRanda (http://www.microrna.org/) databases were used in combination to predict miRNAs that potentially bind to the differentially expressed mRNAs. For differentially expressed lncRNAs, the miRcode database (http://www.mircode.org/) was utilized to predict miRNAs that interacted with them. Additionally, the StarBase database (http://starbase.sysu.edu.cn/) was employed to predict miRNAs that bind to the differentially expressed circRNAs. By integrating the prediction data from these databases, the relevant mRNAs, circRNAs, lncRNAs, and their corresponding miRNAs were imported into Cytoscape software (version 3.8.2) for visualization and construction of the ceRNA network.

### 2.3 Functional enrichment analysis of differentially expressed mRNAs

The R packages “org.Hs.eg.db”,“clusterProfiler”,“enrichplot”, and “ggplot2” were employed to convert the differentially expressed mRNAs from Gene Symbol to entrez ID and perform Gene Ontology (GO) enrichment analysis. Additionally, the R packages “org.Hs.eg.db”, followed by the R packages “clusterProfiler”, “org.Hs.eg.db”, “enrichplot” and “ggplot2”, were utilized for visualization of the GO enrichment analysis results. Furthermore, the Kyoto Encyclopedia of Genes and Genomes (KEGG) enrichment analysis of differentially expressed mRNAs was conducted using KEGG Orthology-Based Annotation System (KOBAS) (http://kobas.cbi.pku.edu.cn/) to investigate their potential roles and pathways they may affect.

### 2.4 Hub gene screening

The gene network information was imported into Cytoscape software for further analysis. The CytoHubba plug-in was utilized to identify hub genes with significant connectivity within the network. CytoHubba was also used to calculate the connectivity scores of each protein node based on its interactions with other nodes in the network. After running the CytoHubba analysis, the top 10 genes with the highest connectivity scores were identified as hub genes.

### 2.5 Hub gene expression analysis

Gene sequencing expression data from OC and normal samples were examined using the exoRBase 2.0 database (http://www.exorbase.org/) online tool. The expression of the top 10 Hub genes was compared between patients with OC and normal controls, and box plots of the corresponding Hub genes were created.

### 2.6 Hub-mRNA immunocorrelation analysis

RNA-Seq expression profiling data and clinical information for patients with OC were retrieved from The Cancer Genome Atlas Program database (https://portal.gdc.com). The R language was used to analyze the RNA-Seq data and determine the infiltration levels of seven types of immune cells (B cells, CD4+ T cells, CD8+ T cells, natural killer (NK) cells, macrophages, endothelial cells, and uncharacterized cells) in tumor tissues. Spearman’s correlation analysis was used to assess the correlation between non-normally distributed quantitative variables.

### 2.7 Single multifactor analysis of clinical characteristics

TIMER (https://cistrome.shinyapps.io/timer/) was employed to assess the clinical significance of OC immune subgroups and adjust for various covariates in a multivariate Cox proportional risk model. Clinical characteristics (age, gender, race, tumor stage, etc.) and the expression of Hub genes were considered confounders. Subsequently, Cox regression analysis was conducted, and the results, including risk ratios and statistical significance, were documented. TIMER was used to generate Kaplan–Meier plots that depicted both immune infiltration and gene profiles to illustrate the disparities in survival outcomes.

### 2.8 Human Protein Atlas (HPA) immunohistochemical analysis

We entered identified key mRNAs in The Human Protein Atlas database (http://www.proteinatlas.org/), selected “Pathology”, selected the cancer type as “Ovarian Cancer”, and determined the relevant antibody in OC for the protein immunohistochemistry experiment to start the analysis.

### 2.9 Statistical treatment

The data was organized using the Perl programming language (version strawberry-perl-5.32.), and data analysis and plotting were done using RStudio (version 4.1.0). Measures were presented as mean ± standard deviation. Statistical tests, including t-tests or ANOVA, were employed to analyze the data. Statistical significance was determined with a corrected *P*-value of < 0.05.

## 3 Results

### 3.1 Data download and variance analysis

**Fig 1:**
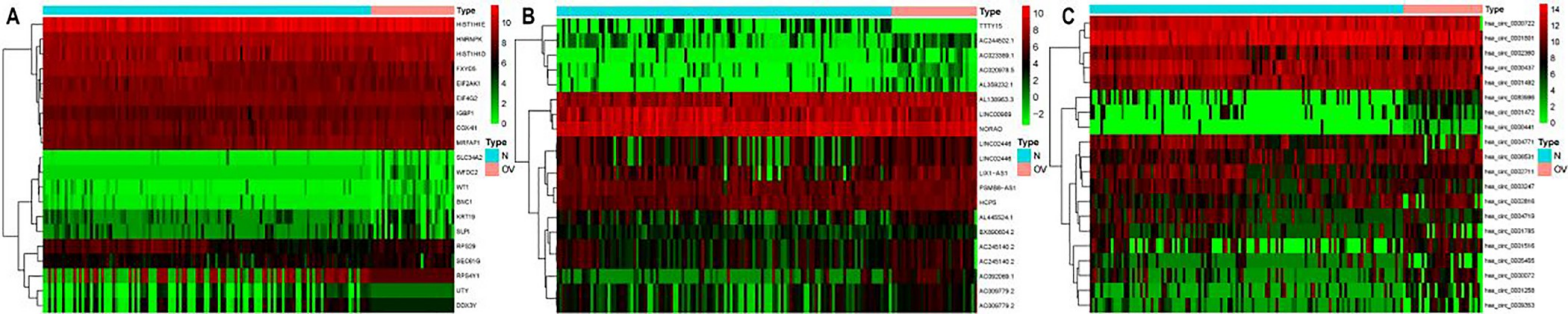
A: mRNA differential expression heatmap; B: lncRNA differential expression heatmap; C: circRNA differential expression heatmap.

In this study, we collected blood exosome sequencing data from 30 patients with OC and 118 healthy individuals using the exoRBase 2.0 database. We also gathered corresponding mRNA, lncRNA, and circRNA expression profiles for further analysis. To identify significant differences in gene expression between patients with OC and healthy individuals, we conducted a differential analysis using the R language. Consequently, we discovered 31 differentially expressed mRNAs, 17 differentially expressed lncRNAs, and 24 differentially expressed circRNAs. We created a heat map to visualize the expression patterns of these differentially expressed genes. Additionally, the top 10 differentially expressed genes were highlighted on the heat map (Fig 1A–1C).

### 3.2 Prediction of miRNAs and construction of related ceRNA regulatory networks

**Fig 2:**
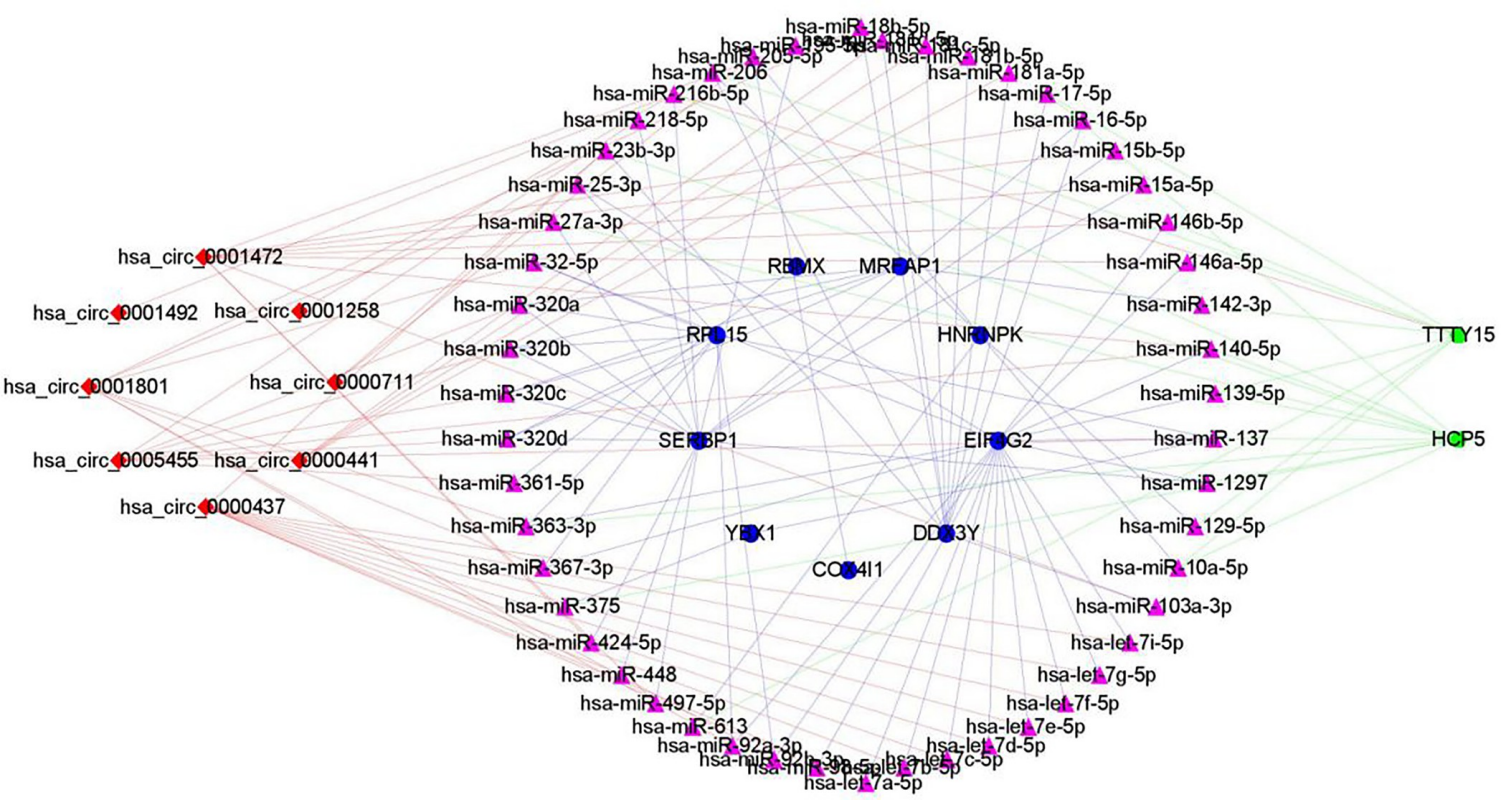
ceRNA network of differentially expressed genes.

In this study, we predicted miRNAs that would interact with the differentially expressed mRNAs (n = 136) using TargetScan and miRanda databases. Further, miRcode database, we identified miRNAs (n = 176) that were expected to bind to the differentially expressed lncRNAs. Additionally, using the starBase database, we identified miRNAs (n = 204) that were expected to bind to the differentially expressed circRNAs. We constructed a ceRNA network using Cytoscape software. The ceRNA network comprises nine mRNA nodes, two lncRNA nodes, eight circRNA nodes, and 51 miRNA nodes (Fig 2).

### 3.3 Enrichment analysis of the differentially expressed KEGG pathway

**Fig 3:**
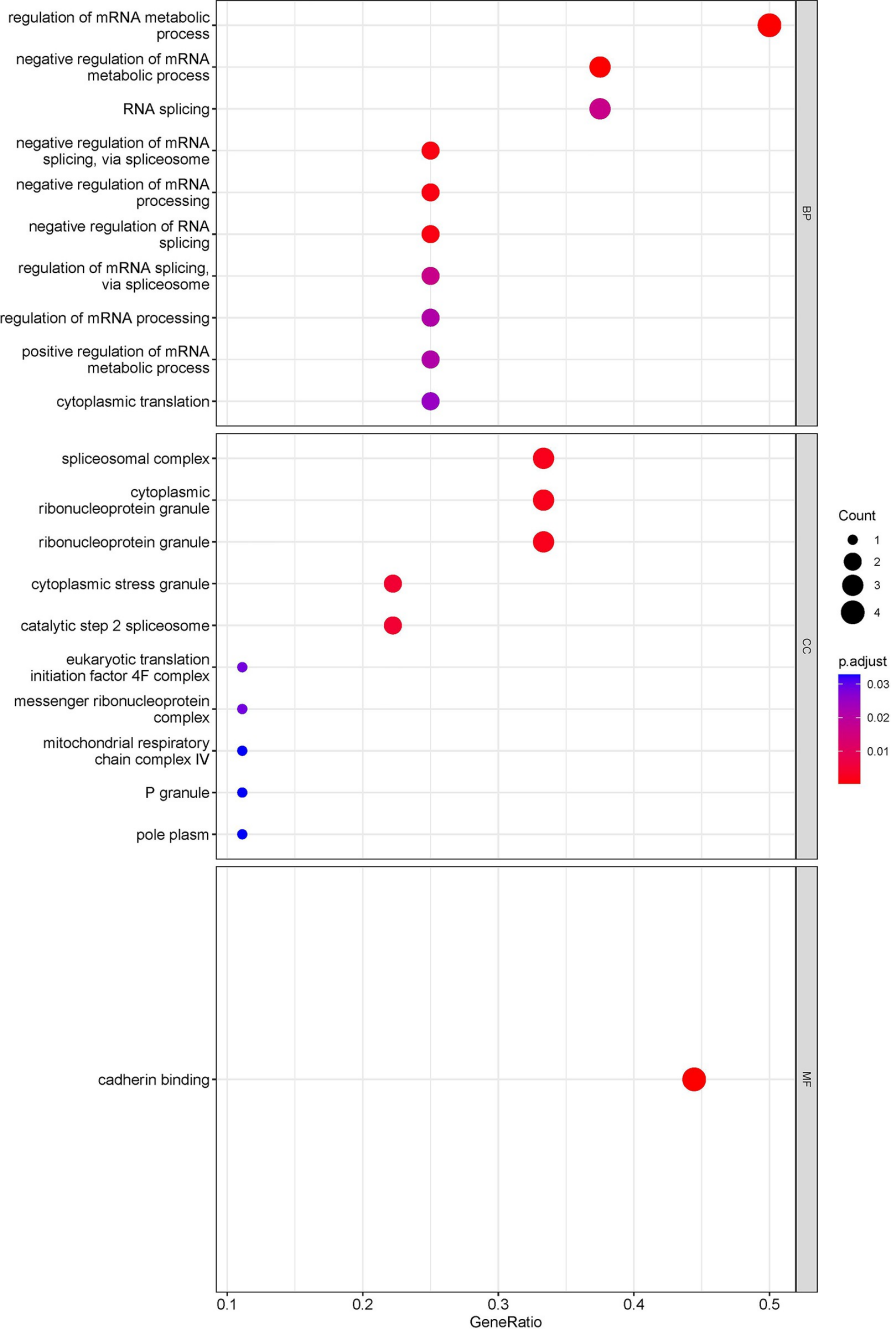
Bubble diagram of GO enrichment analysis.

**Fig 4:**
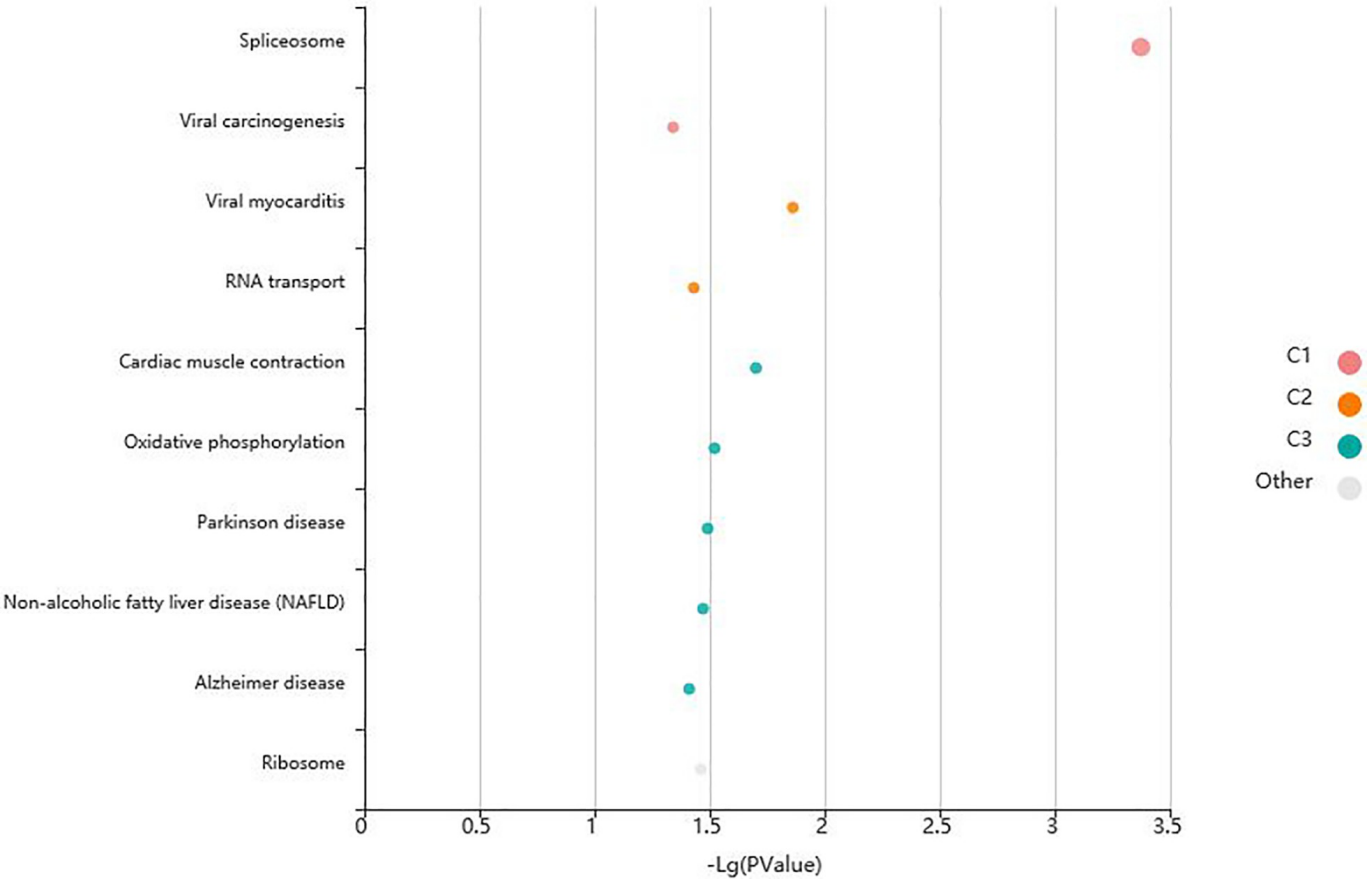
Bubble diagram of KEGG enrichment analysis.

**Fig 5:**
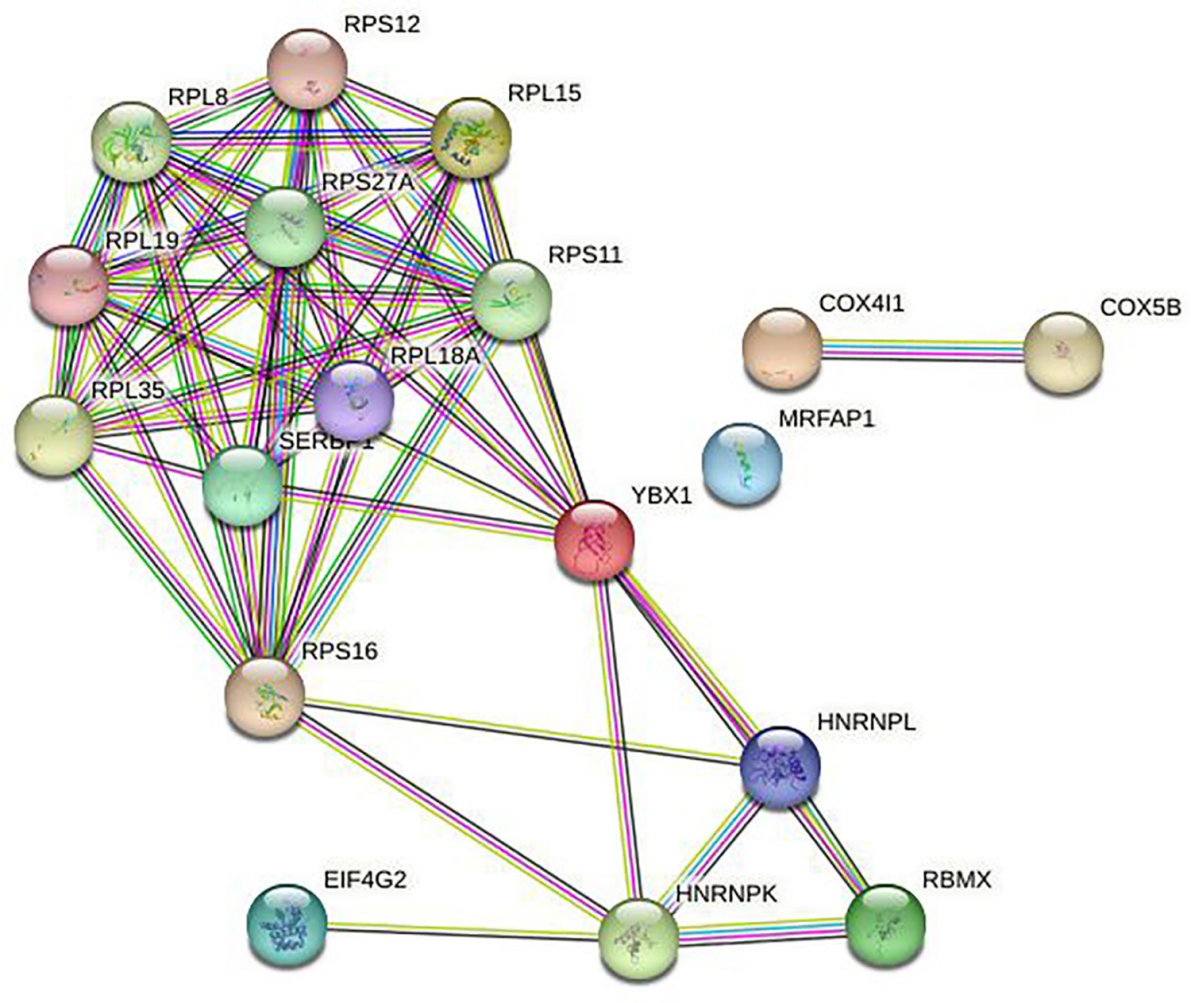
Protein-protein network extracted the mRNA in the ceRNA network.

In this study, we used R packages “clusterProfiler”,“org.Hs.eg.db”, “enrichplot” and “ggplot2” to perform GO enrichment analysis and visualize differentially expressed mRNAs in OC. Additionally, KOBAS (http://kobas.cbi.pku.edu.cn/) was used for KEGG enrichment analysis and visualization of differentially expressed mRNAs. The GO enrichment analysis demonstrated that the differentially expressed mRNAs were significantly enriched in several biological processes, including “regulation of mRNA metabolic process”, “negative regulation of mRNA metabolic process”, “negative regulation of mRNA splicing, via spliceosome”, “negative regulation of mRNA processing”, “negative regulation of RNA splicing”, “spliceosomal complex”, “cytoplasmic ribonucleoprotein”, “cytoplasmic ribonucleoprotein granule”,“ribonucleoprotein granule”, “cytoplasmic stress granule”, “catalytic step 2 spliceosome”, and “cadherin binding” (Fig 3). The KEGG enrichment analysis revealed significant enrichment of differentially expressed mRNAs in two main pathways, namely the “Spliceosome” and ‘Viral carcinogenesis” pathways (Fig 4). In the “Spliceosome” pathway, two genes, *heterogeneous nuclear ribonucleoprotein K* (*HNRNPK)* and *RNA binding motif protein X-linked*, were identified as the most significant genes (Fig 5). To contrast, in the “viral carcinogenesis” pathway, the most significant gene identified was *eukaryotic translation initiation factor 4 gamma 2* (*EIF4G2*) (Table 1).

**Table 1:** Results of KEGG enrichment analysis.

Term	Database	ID	P-Value	Input
**Spliceosome**	**KEGG PATHWAY**	**hsa03040**	**0.000428405**	**HNRNPK|RBMX**
**Viral myocarditis**	**KEGG PATHWAY**	**hsa05416**	**0.013900975**	**EIF4G2**
Cardiac muscle contraction	KEGG PATHWAY	hsa04260	0.019773583	COX4I1
Oxidative phosphorylation	KEGG PATHWAY	hsa00190	0.030310605	COX4I1
Parkinson disease	KEGG PATHWAY	hsa05012	0.032316807	COX4I1
Non-alcoholic fatty liver disease (NAFLD)	KEGG PATHWAY	hsa04932	0.033874634	COX4I1
Ribosome	KEGG PATHWAY	hsa03010	0.03476382	RPL15
RNA transport	KEGG PATHWAY	hsa03013	0.037427015	EIF4G2
Alzheimer disease	KEGG PATHWAY	hsa05010	0.038756162	COX4I1
Huntington disease	KEGG PATHWAY	hsa05016	0.043615753	COX4I1
Viral carcinogenesis	KEGG PATHWAY	hsa05203	0.045377455	HNRNPK

### 3.4 Key gene screening

**Fig 6:**
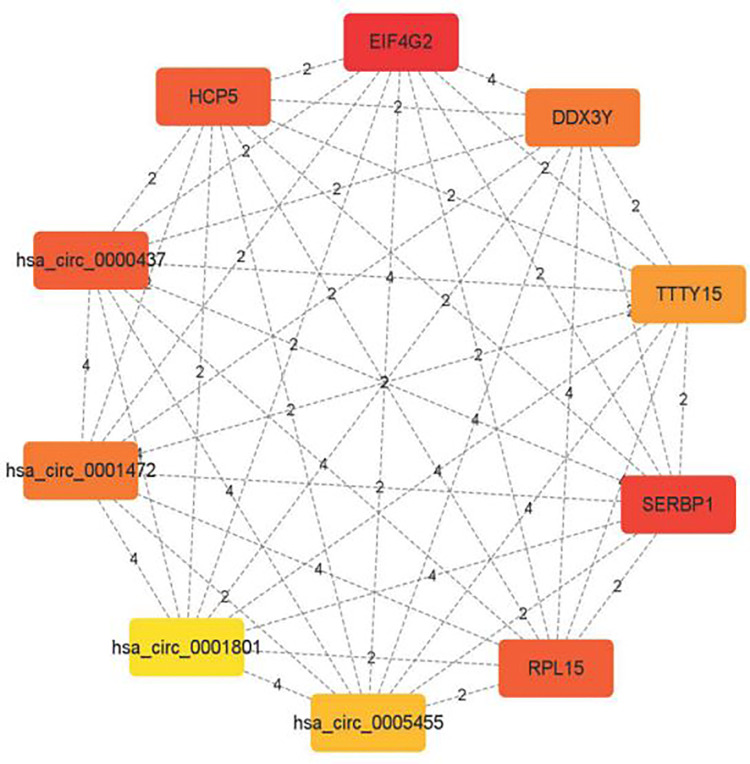
Hub gene network diagram.

Cytoscape software was used to screen the Hub genes with the top 10 scores in the ceRNA network, including the following two lncRNAs, *testis expressed transcript*, *Y-linked 15* (*TTTY15*), and *human leukocyte antigen complex P5* (*HCP5*); four mRNAs, *SERPINE 1 mRNA binding protein 1* (*SERBP1*), *ribosomal protein L15* (*RPL15*), and *DEAD-box helicase 3 Y-linked* (*DDX3Y*); four circRNAs, hsa_circ_0000437, hsa_circ_0001472, hsa_circ_0005455, and hsa_circ_0005455 to construct the corresponding Hub gene network maps. It was suggested that genes closely related to the development of OC were EIF4G2, SERBP1, RPL15, DDX3Y, and HCP5 (Fig 6).

### 3.5 Hub gene expression analysis

**Fig 7:**
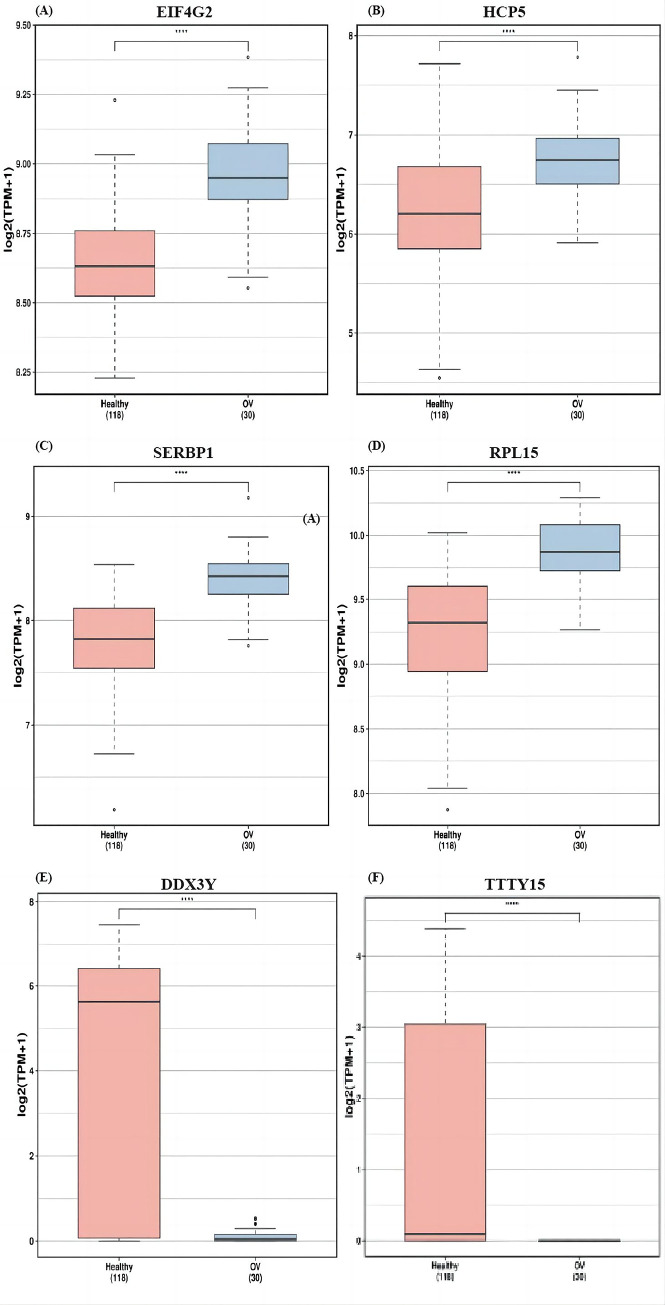
Differentially expressed genes in patients with OC (n = 30) vs. normal controls (n = 118).

Further analysis of EIF4G2, SERBP1, RPL15, DDX3Y, HCP5, and TTTY15 genes using exoRbase online analysis tool demonstrated that EIF4G2, SERBP1, RPL15, and HCP5 were significantly more highly expressed in patients with OC compared to normal controls, while TTTY15 and DDX3Y gene levels were significantly lower in patients with OC compared to normal controls (Fig 7).

### 3.6 Immunocorrelation analysis

**Fig 8:**
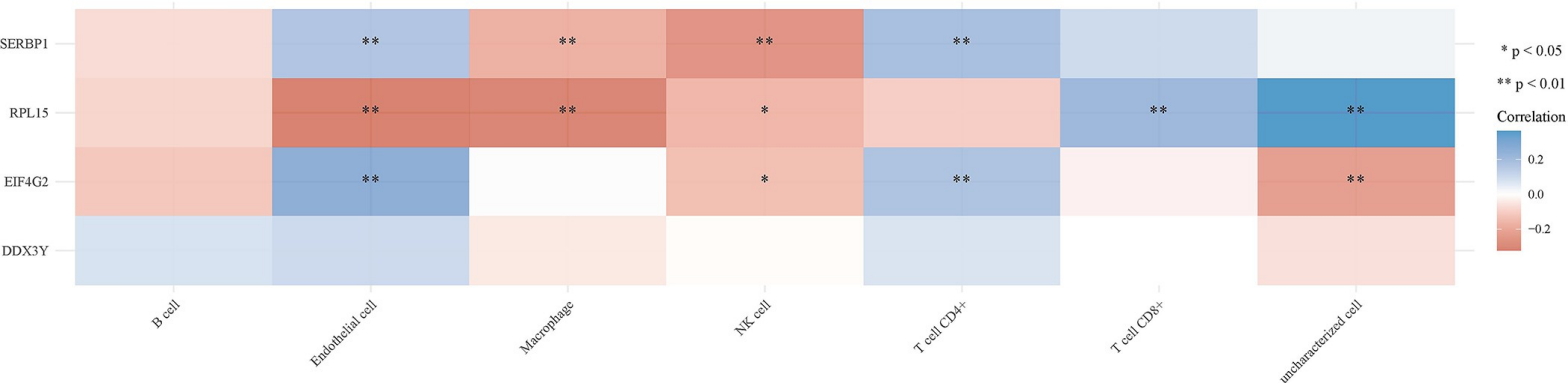
Immunological correlation analysis.

R linguistic analysis of RNA-Seq expression profiling data was performed to detect the association between seven immune cells (B cells, CD4+ T cells, CD8+ T cells, NK cells, macrophages, endothelial cells, and uncharacterized cells) in tumor tissues and EIF4G2, SERBP1, RPL15, and DDX3Y gene immunity. The results demonstrated that SERBP1 was significantly negatively correlated with endothelial cells and CD4+ T cells, and positively correlated with NK cells and macrophages. EIF4G2 was significantly negatively correlated with endothelial cells and CD4+ T cells and positively correlated with uncharacterized cells (Fig 8).

### 3.7 Multi-factor cox analysis and model construction

**Fig 9:**
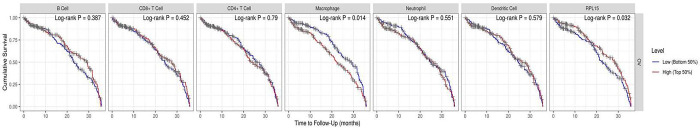
Curve of 3-year survival prediction.

**Fig 10:**
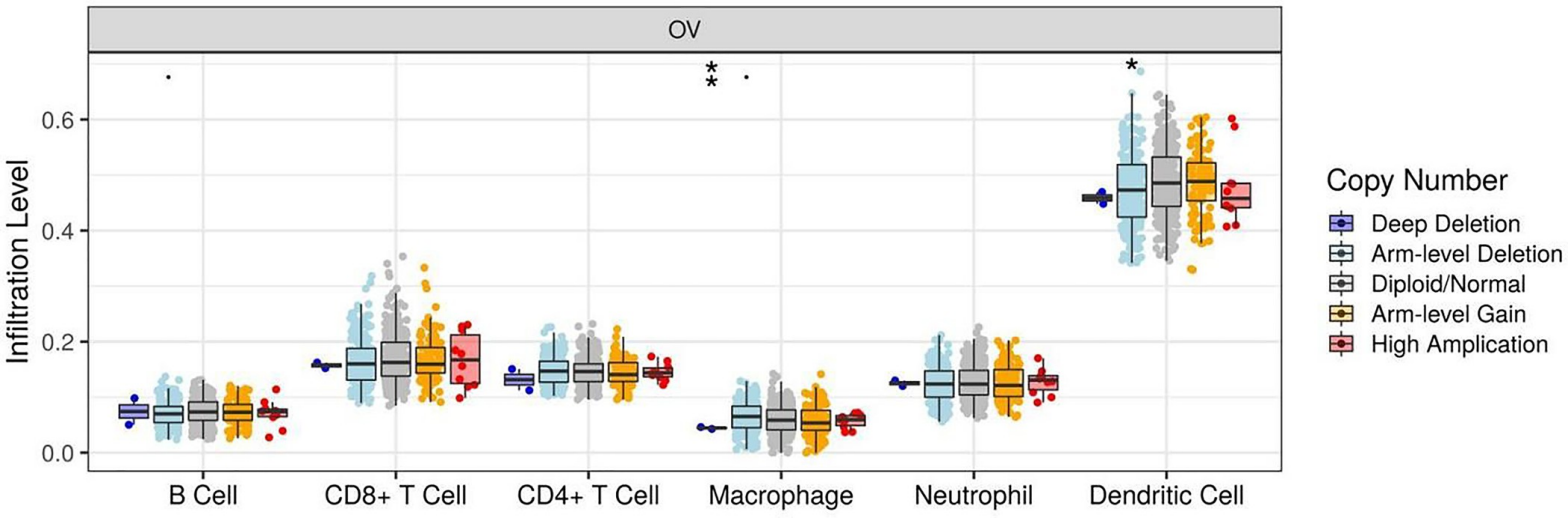
Box plot of the distribution of different copy number states of different immune cells.

The clinical information, including age, race, tumor stage and grade, and tumor purity was preprocessed. Risk scores were obtained according to the model, and multi-factor Cox regression analysis was performed to combine hub genes with B_cell, CD8_Tcell, CD4_Tcell, macrophage, neutrophil, and dendritic cell and construct the corresponding prognostic models. The optimal model was constructed using multifactorial Cox regression analysis (Model: Surv(OC) ~ Age + Race + Purity + B_cell + CD8_Tcell + CD4_Tcell + Neutrophil + Macrophage + Dendritic + RPL15, 437 patients (with 281 deaths). Six factors significantly correlating with survival were obtained-age, purity, CD8_Tcell, CD4_Tcell, neutrophil, and macrophage-based on survival information and significant correlation characteristics derived from multifactorial Cox analysis (P < 0.05), and the curves of 3-year survival prediction were plotted using RMS in the R package. The survival prediction curves demonstrated that the prognostic factors associated with 3-year survival in paatients with OC were macrophage and RPL15 (Fig 9). The copy number variation of PRL15 in different immune cells in OC was also determined (Fig 10).

### 3.8 HPA immunohistochemical analysis

**Fig 11:**
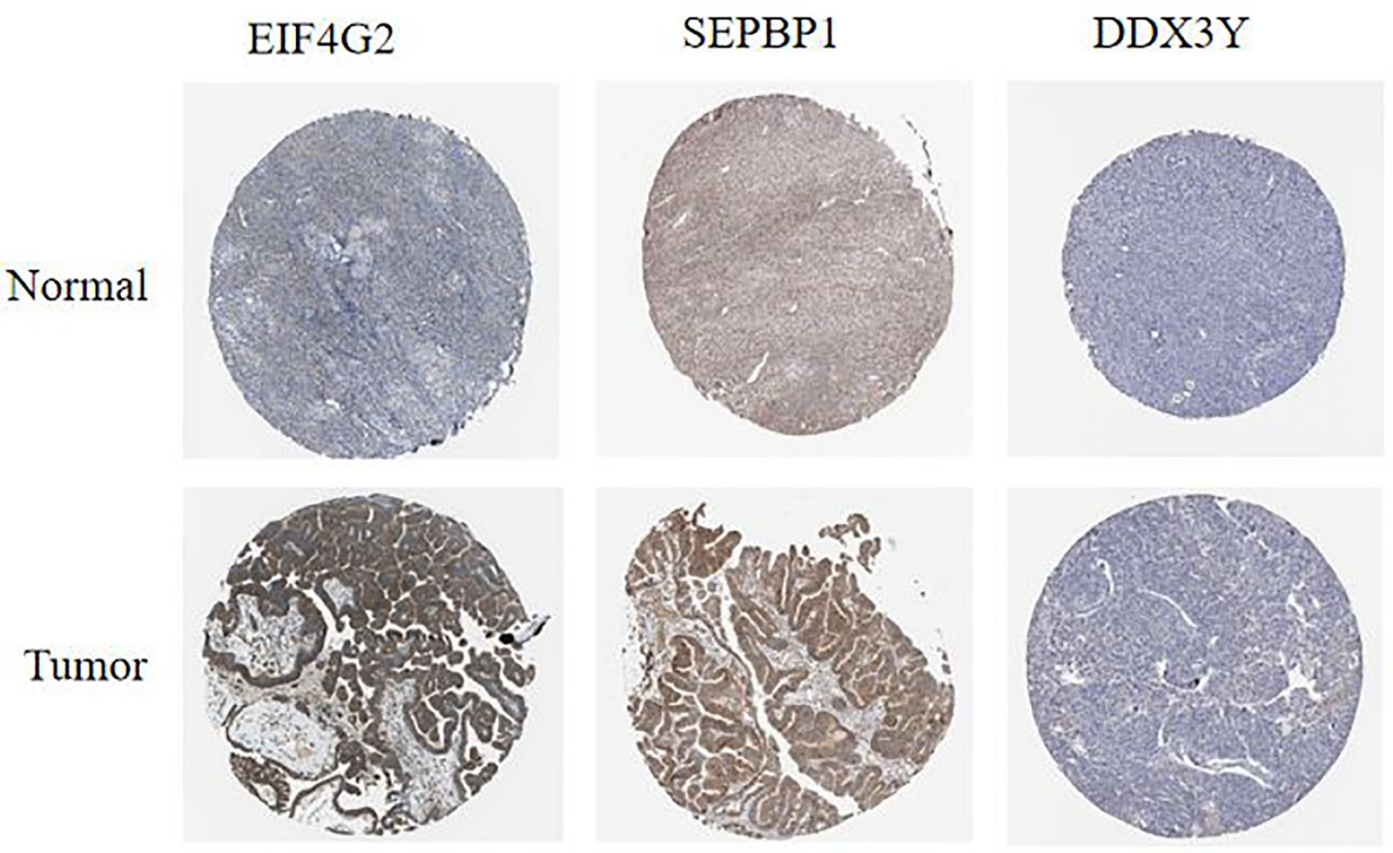
Human Protein Atlas (HPA) protein expression.

The HPA online tool was used to conduct an immunohistochemical analysis of three genes (EIF4G2, SERBP1, and DDX3Y) in OC tissues and normal ovarian tissues. The findings revealed significantly elevated protein expression levels of EIF4G2 and SERBP1 in tumor tissues compared to normal ovarian tissues. Similarly, DDX3Y protein expression was significantly higher in tumor tissues than in normal ovarian tissues. Conversely, DDX3X protein expression was lower in both normal ovarian tissues and cancer tissues, however, the difference was not statistically significant. (Fig 11).

## 4 Discussion

OC is a prevalent malignancy affecting the female reproductive system. Currently, the primary clinical treatments for OC are limited, including surgery, chemotherapy, or targeted drug therapy. Although a small number of patients may achieve a cure through surgery alone, most of them usually require comprehensive treatment, such as a combination of surgery and chemotherapy. The early symptoms of OC are insidious, and most patients have local or distant transmission by the time of diagnosis [15]. Therefore, early diagnosis and treatment are significant for OC, and discovering new targets for OC diagnosis and treatment emerged as a challenge that needs to be overcome. Exosomes are rich in bioactive molecules, including DNA, mRNA, miRNA, and proteins, making them a popular research area recently. Exosomes are of great research value as they transmit their bioactive components across cells to carry out the crucial task of intercellular communication [16]. It has been demonstrated that exosome-derived lncRNAs are involved in mediating intercellular signaling that plays an essential role in tumorigenesis and development [17]. Recently, the ceRNA hypothesis has been reported to be a valuable framework for understanding tumor pathogenesis and has paved the way for tumor diagnosis and treatment research. Non-coding RNAs, acting as ceRNAs, have emerged as a major focus of investigation in various diseases.miRNAs, as crucial post-transcriptional regulators, can have their activities modulated by lncRNAs. Additionally, the expression of target genes can be regulated by lncRNAs and circRNAs, which can function as ceRNAs and compete with miRNAs to alter mRNAs, ultimately regulating the protein levels of coding genes. The lncRNA-miRNA-mRNA ceRNA regulatory network has been reported to play significant roles in gastric, breast, pancreatic, and liver cancers [18–20]. The application of the ceRNA hypothesis and its validation in diverse cancer types serve as a valuable reference for exploring the mechanisms of the ceRNA regulatory network in OC. Understanding the ceRNA interactions in OC may help identify the potential therapeutic targets and diagnostic markers, contributing to improved management and treatment outcomes for patients with OC.

In this study, we uesd the exoRBase database to analyze the sequencing data of peripheral blood exosomes from patients with OC and healthy individuals. Through this analysis, we identified 31 differentially expressed mRNAs, 17 differentially expressed lncRNAs, and 24 differentially expressed circRNAs. Subsequently, we constructed a ceRNA network based on shared miRNAs using Cytoscape software. The network comprised two lncRNA nodes, nine mRNA nodes, eight circRNA nodes, and 51 miRNA nodes. These include “mRNA metabolic process,” “negative regulation of mRNA metabolic process,” “negative regulation of mRNA splicing, via spliceosome,” “negative regulation of mRNA processing,” “negative regulation of RNA splicing,” “spliceosomal complex,” “cytoplasmic ribonucleoprotein granule,” “ribonucleoprotein granule,” “cytoplasmic stress granule,” “catalytic step 2 spliceosome,” “cadherin binding”. KEGG analysis results were mainly enriched in Both GO and KEGG were concentrated in spliceosome-related pathways, and surface spliceosomes were closely linked to the development of OC. The Cytoscape software screened the top 10 Hub genes, including two lncRNAs, four mRNAs, four circRNAs. Compared with those in normal controls, EIF4G2, SERBP1, RPL15, and HCP5 were significantly more highly expressed in patients with OC, whereas TTTY15 and DDX3Y genes were horizontally less expressed in vivo.

Immunocorrelation analysis demonstrated that SERBP1 was significantly negatively correlated with endothelial cells and CD4+ T cells and significantly positively correlated with macrophages and NK cells. RPL15 was significantly positively correlated with macrophages and endothelial cells, and negatively correlated with CD8+ T cells and uncharacterized cells. EIF4G2 was significantly negatively correlated with endothelial cells and CD4+ T cells and positively correlated with uncharacterized cells. The clinical data including age, race, tumor stage and grade, and tumor purity were preprocessed. The risk score was derived from the model. Multi-factor Cox regression analysis was performed to combine hub genes with B_cell, CD8_Tcell, CD4_Tcell, macrophage, neutrophil, and dendritic cells and construct the corresponding prognostic models. The optimal model was constructed by multifactorial Cox regression analysis (Model: Surv(OC) ~ Age + Race + Purity + B_cell + CD8_Tcell + CD4_Tcell + Neutrophil + Macrophage + Dendritic + RPL15 437 patients (with 281 dying)). Six factors that significantly correlated with survival were obtained age, purity, CD8_Tcell, CD4_Tcell, neutrophil, and macrophage based on survival data and significant correlation characteristics (P < 0.05) derived from multifactorial Cox analysis, and 3-year survival was plotted using RMS in the R package. The survival prediction curves demonstrated that the prognostic factors associated with the 3-year survival of patients with OC were macrophage and RPL15. Immunohistochemical analysis revealed significantly higher expression levels of EIF4G2, SEPBP1, and DDX3Y in tumor tissues.

The ribosomal large subunit protein RPL15 belongs to the Pfam00827 family, and its cDNA contains 759 nucleotides and can encode 204 amino acids with a protein molecular mass of approximately 24 × 103 (24 kDa) [21]. Since RPL15 was first cloned and identified in lobster [22] and petunia [23], respectively, numerous studies have focused on its biological role and discovered that RPL15 is also closely related to various tumors and other diseases [24]. Wang et al. [25] observed that the RPL15 protein was overexpressed in esophageal cancer cells and involved in the efficient cleavage of the ITS1 site in 47S pre -rRNA, thus, further confirming the involvement of this protein in the processing of ribosomal RNA. Kashuba et al. [26] identified a group of eight biomarkers with significant potential for detecting both early and advanced OC in their study. These biomarkers are *nuclear factor kappa B inhibitor interacting Ras-like 1*/RPL15, *thyroid hormone receptor beta*, *ribosomal protein S3* (*CTD small phosphatase like*), *IQ motif and sec7 domain ArfGEF 1*, *neurobeachin like 2*, *Zic family member 4*, LOC285205, and *forkhead box P1*. Among these biomarkers, RPL15 has been highlighted as a potential diagnostic marker for OC.

HCP5, formerly known as an oncogene, encodes a 132-amino acid small protein called HCP132-5aaa. Xiao et al. [27] demonstrated that HCP132-5aa promotes TNBC growth by modulating GPX4 and subsequently inhibiting ROS levels, thus, inhibiting ferrozois. A study by Liu et al. [28] revealed that HCP5 increases *pleiomorphic adenoma gene-like 2* expression by activating the Wnt/β-catenin signaling pathway via the sponge mir-128-3p, thereby, promoting the multiple myeloma cell proliferation and tumor formation. Tan et al. [29] demonstrated that lncRNA of HCP5 plays a significant role in promoting osteosarcoma (OS) proliferation, migration, and invasion via the miR-29b-3p/LOXL2. Their study revealed that HCP5 can upregulate *lysyl oxidase-like 3* expression by targeting miR-2b-29p, thereby promoting OS cell proliferation, migration, and invasion. EIF4G2 proteins are also known as DAP5, Nat1 and p97. Currently, findings by Shestakova ED et al. suggest that EIF4G2 is a helper protein that facilitates elusive translation mechanisms under rare conditions, andits specific role under normal conditions remained unclear until recently [30]. The study conducted by Liu et al. demonstrated that EIF4G2 is upregulated in gastric cancer (GC). Elevated EIF4G2 expression was reported to be associated with an unfavorable prognosis for patients with GC. Moreover, the study suggested that EIF4G2 expression may play a role in regulating tumor immune cell infiltration [31].

## 5 Conclusion

In this study, we identified exosomal RNAs associated with OC development. Through our analysis, we screened and identified differentially expressed mRNA, lncRNA, and circRNA genes. Additionally, we conducted a KEGG enrichment analysis to gain insights into the functional pathways involved in OC. Based on the results, we constructed a ceRNA network, which enabled us to identify key genes involved in the development of OC. These genes may serve as potential targets for future research focusing on the understanding and treatment of OC.

Nevertheless, this study has certain limitations that must be addressed. The small sample size of included exosomes may introduce potential bias in the screening of lncRNAs, circRNAs, mRNAs, and miRNAs. To overcome this limitation, further validation with large-scale clinical trials and more extensive sample sizes is necessary to ensure the robustness and generalizability of the findings.

## Abbreviations

ceRNA: competing endogenous RNAs
OV: ovarian cancer
GO: gene ontology
KEGG: Kyoto Encyclopedia of Genes and Genomes
lncRNA: long noncoding RNA
mRNA: messenger RNA
miRNA: micro RNA
